# Enhancement of Agricultural Processed By-Products: Qualities Analysis of Fermentation Method in Gradient Salt Adding Treatment of Tuna Cooking Juice with Black Bean Koji Added

**DOI:** 10.3390/foods9030320

**Published:** 2020-03-10

**Authors:** Jhih-Ying Ciou, Lu-Sheng Hsieh, Tzu-Tai Lee, Chang-Wei Hsieh

**Affiliations:** 1 Department of Food Science, Tunghai University, No. 1747, Sec. 4, Taiwan Boulevard, Xitun District, Taichung 40704, Taiwan; lshsieh@thu.edu.tw; 2 Department of Animal Science, National Chung Hsing University, 145 Xingda Rd, Taichung 402204, Taiwan; ttlee@dragon.nchu.edu.tw; 3 Department of Food Science and Biotechnology, National Chung Hsing University, 145 Xingda Rd, Taichung 402204, Taiwan; welson@nchu.edu.tw

**Keywords:** fish sauce, gradient salt-adding method, tuna cooking juice, by-product, koji

## Abstract

Fish sauce is popular for fermenting food in Southeast and Eastern Asia, while black bean is used to ferment condiments in Taiwan. Researchers have recently investigated the use of fish and black bean sauce in places where combining both fish and black bean is rare. This study was focused on fish sauce made from concentrated tuna cooking juice mixed with black bean koji. The experiment was divided into two stages. In the pre-fermentation stage, a suitable fermentation time with no salt added was determined. In the later fermentation stage, two preformatted samples of 4 and 7 days were added to salt water at 20 °Bé. In the pre-fermentation stage, the results show that the protease activity increased as time passed, but the pH value decreased. The highest browning degree was achieved after 120 days. In the later fermentation period, the total nitrogen contents for both experimental groups of 4 days and 7 days reached up to twice that of soy sauce. The total nitrogen content increased with time. In addition, the level of ammonia nitrogen increased from 0.08 to 0.15 g/dL in the first month. In conclusion, a new flavor of fermented sauce was produced in a shorter time and more effectively by combining tuna cooking juice and black bean.

## 1. Introduction

Fermented foods, including soy sauce and fish sauce, are popular Chinese sauces in Southeast and Eastern Asia [1,2,3]. Soy sauce is made by fermenting soybeans, while fish sauce is made by fermenting fish. In Taiwan, a traditional fermented sauce made with black bean called “inyu” uses a similar process but contains different ingredients than soy sauce [4]. 

In this study, we followed the traditional process of making fish sauce by using fresh fish (meat and viscera) premixed with a suitable amount of salt in a vat and then exposing it to a ventilated and sunny place [5]. In order to enhance the economic value of processing by-products and reduce the environmental problems caused by waste from processing, we investigated low-salt fish sauce production through utilization of by-products [6]. Using this method, the fish protein could be decomposed via the richness of peptides and amino acids by autolysis of its own proteases [7]. To obtain a certain flavor, it was necessary to brew the sauce for a long time—about six months to one year [8].

A salt gradient is often used to shorten the fermentation period through adjustment of the salt added. Fermented fish sauces have different names all over the world, such as jeotkal in Korea, uoshoyu in Japan, bakasang in Indonesia, momoni in Ghana, feseekh in Egypt, atis in the Philippines, nuoc-man in Vietnam and Cambodia, and plaa-som in Thailand [9]. Even in Europe, there are so-called anchovy sauce products. Due to the strong smell of fish sauce, it is known as “yu-lu” in Chinese and has been poorly accepted by the people of Taiwan in the past [10]. 

In previous studies of fish sauce, efficient processing and the reduction of fermentation time could be achieved by applying enzymes from fish viscera [11] and halophilic marine bacteria [12]. Moreover, it was obviously effective to suppress the tangy, fishy smell by mixing sauce or extra additions (alcohols) into the fish sauce [13,14] and to improve the flavor by using beans with wheat koji and fish in the vat [15]. In Taiwan, some researchers have also focused on the most suitable conditions of beans with wheat koji and fish for brewing fish sauce [16], and some have even utilized the viscera of bonito, soy koji, or red koji as the source of decomposing enzymes, in order to explore the feasibility of using the head and viscera of bonito and other wastes, as the raw materials of fish sauce [17]. For faster fermentation conditions, utilization of minced capelin with the small intestines of cod, including rich trypsin and chymotrypsin, for six months could produce fish sauce [16].

Moreover, Guillerm added fresh pineapple juice during the processing of fish sauce in order to shorten the fermentation time and increase the storage time under a lower pH value [18]. Fisheries and aquatic products processing industries are prospering, as Taiwan is an island. Because many by-products are often produced by using aquatic raw materials during processing, aquatics can produce lots of cooking juices during processing and disposal. Tuna represents one of the most important fisheries in Taiwan, producing over 300,000 tons with a value of approximately NTD 31 billion every year [19]. In fact, tuna cooking juices are an effective by-product after processing, as canned tuna and its cooking juices include considerable amounts of flavor [20]. Canned tuna, for example, will produce 160 kilograms of cooking juices per ton of raw materials. These cooking juices include rich flavor substances, i.e., extractive components, and include about 4.1% protein [21]. They can be described as cheap, fine flavor sources [21]. 

Black bean is a nutritious food that contains protein, vitamin E, isoflavones, carotenoids, anthocyanins, and more [22]. Additionally, black beans can be prepared as black bean koji by growing fungi on steamed black bean substrate, thus producing a condiment that is popular in Taiwan [23]. There are many studies regarding black bean koji and tuna cooking juice; however, the combination of black bean koji with tuna cooking juice has not been reported. Therefore, this study aimed to produce a flavorful sauce by combining tuna cooking juice and black bean koji and to enhance the economic value of tuna cooking juice by turning it into a new product. This study analyzed the quality of the resulting sauce. 

## 2. Materials and Methods

### 2.1. Raw Materials

Tuna cooking juice was directly collected as a by-product from the canned marine products industry and then concentrated to 40% protein content for further analysis. Black bean koji (*Aspergilus oryzae*) was grown after 4 days and provided by Da Shan Equipment Raw Material, Taoyuan, Taiwan R.O.C.

### 2.2. Fish Sauce Samples Preparation

The steamed black beans were pre-processed by dipping, draining, steaming (120 °C, 25 min), and then cooling, before being mixed with the roasted wheat flour (steamed black bean/roasted wheat flour = 1:0.2–0.4). This mixture, inoculated with the spore of *Aspergillus oryzae*, could produce koji after 4 days of incubation. Before brewing, concentrated tuna juice with koji was added to different percentage salt solutions for the adjustment of protein concentration. The content of salt was controlled at 20 °Bé during brewing.

### 2.3. Collection of Liquid

After fermentation and incubation for the designated time, the samples were thoroughly mixed by shaking the tanks. Separate samples were filtered through a Shuang Quan filtered paper (Model 102). The filtrate was used for analysis.

### 2.4. Measurements of Protease Activity

The activity of neutral protease was measured according to Anson (1938) [24]. A total of 12 mL of crude enzymes were added into 48 mL of 2% azocasein solution (pH 7.5) and incubated at 37 °C for 60 min. After adding 60 mL of 10% trichloroacetic acid (TCA) to stop the reaction, the samples were settled for 20 min and then centrifuged at 6000× *g* for 10 min. A total of 20mL of 1.8 N NaOH was added to 80 mL of the supernatants, and then, the absorbance was measured at 420 nm. One unit of protease activity was defined as the amount of enzyme that could cause 0.1 increments of the absorbance at 420 nm within 60 min of the reaction at 37 °C.

### 2.5. Components Analysis

The chemical composition of the sample was determined according to the Association of Official Analytical Chemists (AOAC) method [25]. Crude protein content was determined by the Kjeldahl method, with a conversion factor of 6.25. Crude fat content was measured by extracting the sample in a Soxhlet extraction system using ether. Moisture content was measured by calculating the difference in the sample weight before and after drying at 105 °C for 8 h. Ash was estimated by heating the sample at 600 °C to a constant weight.

### 2.6. Chemical Analysis

Total nitrogen was determined according to the procedure of AOAC [25]. The pH was determined using a pH meter (HM-5 S; TOA Electric Industrial Co. Ltd., Tokyo, Japan). Salt content of the samples was determined according to the AOAC procedure [25]. Formol nitrogen content, a convenient index of the degree of protein hydrolysis, was determined by using the titration method as described [26]. The samples were analyzed for amino nitrogen using a variation of the AOAC method [25]. Amino nitrogen was calculated based on the formol and ammonia nitrogen contents as follows: amino nitrogen content = formol nitrogen content—ammonia nitrogen content. The content of reducing sugar was determined by the DNS method [27]. The content of reducing sugar was calculated based on the standard curve constructed, using glucose as the standard.

### 2.7. Physical Analysis

Non-enzymatic browning was determined using the method of Gomez [9]. Prior to analysis, a sample (5 mL) was extracted in 50 mL of 50% (*w/v*) ethanol with continuous stirring for 1 h. The extract was filtered using filter paper (Gellman Laboratory–Pall, Ann Arbor, MI, U.S.A.). The filtrate was then subjected to the absorbance measurement at 420 nm using a spectrophotometer (Model U2001, Hitachi Co., Tokyo, Japan).

### 2.8. Statistical Analysis

Duncan’s Multiple Range Test was employed to determine the significance of difference among treatments. For each treatment, triplicate measurements were taken. Values were considered to be significantly different when *p* < 0.05.

## 3. Results and Discussion

### 3.1. Studies on the Most Suitable Fermentation Time with Different Salt Contents and Composition Analysis

Currently, most tuna cooking juice, the by-product from the canned marine products industry, is directly discarded. This treatment is a waste because of the high content of protein (4.05%) in tuna cooking juice (Table 1). A protein content of 39.23% and moisture content of 45.2% in tuna cooking juice were obtained after concentrating. A protein content of 40% in concentrated tuna cooking juice is necessary for facilitating delivery and then processing. 

**Figure 1:**
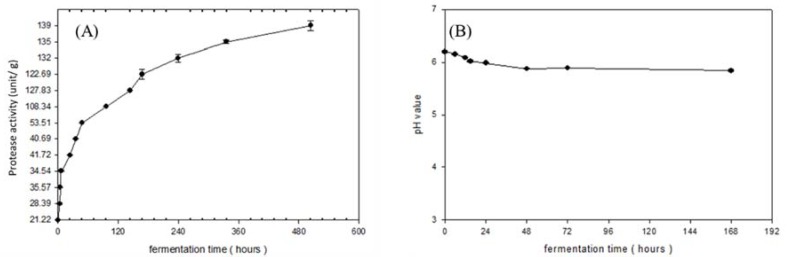
The fermentation time of the protein decomposing (**A**) enzyme activity; and (**B**) pH value changes.

Because a slight decrease of protease activity might be due to poor stability in high salt conditions, the entire fermentation process was assigned two steps. The first stage directly used black bean koji without salt. The utilization of protease from black bean koji during the control of optimal temperature (50 °C) for proteolysis can help to hydrolyze protein in tuna cooking juice. After the first stage (pre-fermentation), salt was added in preparation for fermentation and ripening in the second stage. As Figure 1A shows, the protease activity increased rapidly before 96 h, but slowly after 96 h. According to the results of the sensory evaluation, better flavor was obtained after 4–7 days of fermentation. Therefore, products after 4 and 7 days of fermentation were collected for further analysis and characterization, because time control of fermentation is an important factor in determining the quality of the final product.

According to the result of Figure 1B, the pH continuously decreased down to 5.82 during fermentation. Changes in pH were dependent on the balance of microflora between yeast and lactic acid bacteria. However, at pH 5 to 6, the dominant strain, yeast, could suppress the growth of lactic acid bacteria under aerobic or anaerobic environment [28]. This phenomenon suggests that the growth of microorganisms and the richness of amine in aquatic products can influence the reducing pH, which was similar to that obtained by [29].

### 3.2. Changes in Fish Sauce after Fermentation and in the Period of Maturity

#### 3.2.1. Changes in Browning during the Fermentation Period

**Figure 2:**
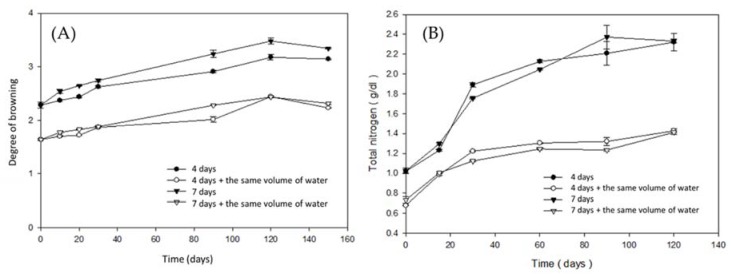
Changes in (**A**) browning during the brewing period of fish sauce of each group; (**B**) total nitrogen during the brewing period of fish sauce of each group (—●—: Group A; —○—: Group B; —▼—: Group C; —△—: Group D).

As shown in Figure 2A, the maximum degree of browning was shown by each sample at 120 days. Because the processing of tuna cooking juice is achieved at high temperatures, the pigment could be produced by this stage and then continuously accumulated during fermentation. This pigment is mainly melanoidin and is usually generated during the brewing of soy sauce [30]. The pigment is caused by Maillard reaction (nonenzymatic browning reaction) [31,32]. Yaylayan (1992) indicated that the rapid formation of melanoidins occurs via the high pressures and temperatures of brewing reactions [33]. Maga and Kim (1989) considered that the existence of reducing sugar and amino acids in food could lead to the follow-up development of melanoidins [30].

#### 3.2.2. Changes in Total Nitrogen during the Fermentation Period

Total nitrogen can be used to classify the quality of soy sauce in Taiwan [34]. According to the national standard of Taiwan, the total nitrogen content should be no less than 1.4 g/100 mL of soy sauce [35]. As shown in Figure 2B, the total nitrogen content of each group exhibited an increasing trend. After 120 days of fermentation, groups A and C were 1.7- and 1.8-fold higher than the national standards for soy sauce in Taiwan, respectively. The total nitrogen content in liquid is also one of the most important quality factors for fish sauce and is used as an indicator to determine the price of fish sauce in Thailand [1,30,36]. The total nitrogen content is composed of protein nitrogen and non-protein nitrogen compounds, such as free amino acids, nucleotide, peptides, ammonia, urea, and trimethylamine oxide, and is used as a contributor of specific aroma and flavor.

#### 3.2.3. Changes in Formaldehyde Nitrogen, Ammonia Nitrogen, and Amino Nitrogen during the Fermentation Period

**Figure 3:**
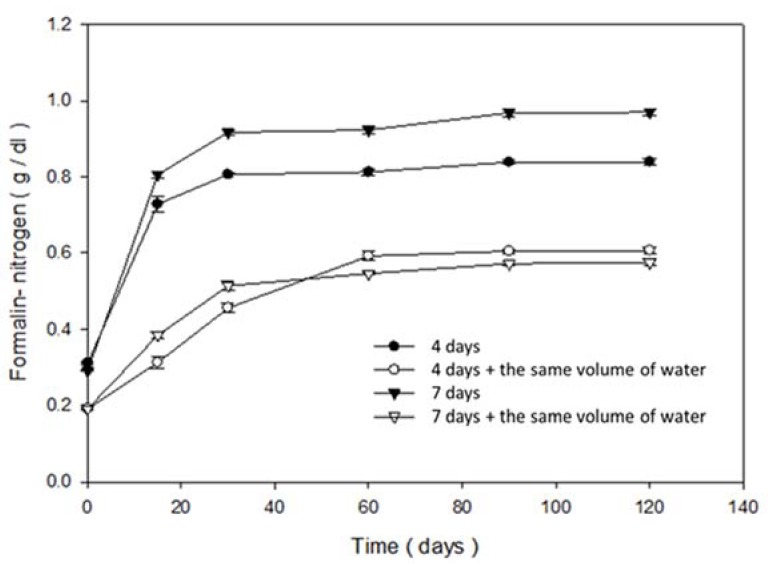
Changes in formaldehyde nitrogen during the brewing period of fish sauce of each group (—●—: Group A; —○—: Group B; —▼—: Group C; —△—: Group D).

There was a similar trend of gradual increase in formaldehyde nitrogen contents over 120 days of fermentation (Figure 3). The highest content of formaldehyde nitrogen in groups A and C was 0.08 and 0.88 g/100 mL, respectively. Because formaldehyde reacts with amino acids and can liberate one H^+^ ion from the amino group and be potentiometrically titrated with a sodium hydroxide solution, formaldehyde nitrogen content was useful for measuring the level of total free amino acids and considered as a convenient index of the degree of protein hydrolysis.

**Figure 4:**
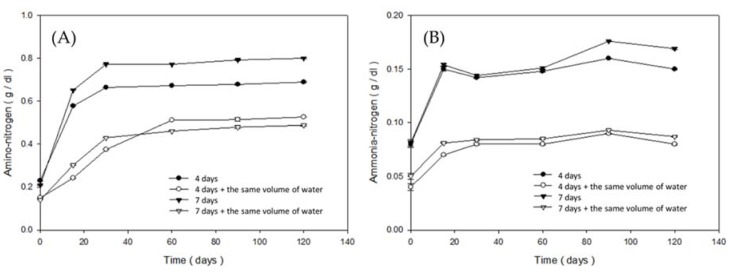
Changes in (**A**) amino nitrogen during the brewing period of fish sauce of each group; (**B**) ammonia nitrogen during the brewing period of fish sauce of each group (—●—: Group A; —○—: Group B; —▼—: Group C; —△—: Group D).

As Figure 4A illustrates, the amino nitrogen content in the fish sauce over 120 days of fermentation was approximately higher than 0.5 g/100 mL. Amino nitrogen contents must not be less than 0.56 g/100 mL, according to national standards in Taiwan [35]. Groups in this study contained amino nitrogen contents of over 0.6 g/100 mL. Klomklao (2006) indicated that amino nitrogen should be considered as nitrogenous compounds from small hydrolysates, particularly amino acids, and that they should be the primary amino group in fish sauce [10]. 

The ammonia nitrogen content of all groups over 120 days of fermentation is shown in Figure 4B. The increased ammonia nitrogen content could be due to the breakdown of soluble protein and peptides into free amino acids and volatile nitrogen by the hydrolysis of fish enzymes during fermentation. As we know, aquatic food is easily decomposed during storage. The composition of putrefaction includes a variety of amines and ammonia. Tungkawachara (2003) indicated that ammonia nitrogen is suitable as a relational index of fish sauce for further understanding its quality and character [36]. 

## 4. Conclusions

In conclusion, a new flavor of fermented sauce was produced by combining tuna cooking juice and black bean koji. Meanwhile, the results of the quality analyses show that the fermentation process became shorter and more effective by introducing gradient salt treatment. Moreover, this provides some benefits, including lowering environmental pollution, utilizing rich protein from tuna, and introducing a new flavor of fermented sauce. In addition, gradient salt treatments significantly enhanced flavor and increased decomposition during processing. Therefore, the resulting fish sauce met the high quality standards of soy sauce and can potentially be used as a marine by-product in the food industry.

## Figures and Tables

**Table 1:** The components of tuna cooking juice.

	Moisture (%)	pH	Crude Lipid (%)	Crude Protein (%)	Ash (%)
Cooking juice (original)	94.07	6.21	0.39	4.05	0.87
Cooking juice (concentrate)	45.2	6.2	3.8	39.23	5.5
